# The Vagaries of Blood-Pressure

**Published:** 1907-06-22

**Authors:** Leonard Williams

**Affiliations:** Physician to the French Hospital, Assistant Physician to the Metropolitan Hospital


### Hospital Clinics.

### THE VAGARIES OF BLOOD-PRESSURE.

By LEONARD WILLIAMS, M.D., M.R.C.P., Physician to the French Hospital, Assistant Physician to the Metropolitan Hospital.

(Lecture delivered at the Medical Graduates' College.)

In my previous lectures * I confined myself to the consideration of such abnormalities in blood-pressure as are both generalised and more or less sustained. I propose to-day to look at the matter from the point of view of the results of alterations in blood-pressure which are local or transient only. I have already insisted, though it is impossible too strongly to insist, upon the central physiological fact which must always be kept in mind if we would understand the phenomena presented by the variations of the blood-pressure, and in order to make the subject of to-day sufficiently clear it is necessary that I should once more refer to it. One of the principal objects, if, indeed, it be not the main object, of the circulation, is to supply all parts of the periphery with oxygenated blood. Now, if this purpose is to be effected, it is obvious that the pressure of the fluid within the vessels must be maintained at or above a certain level. If the stream becomes too sluggish, certain parts of the periphery fail to receive their nourishment, and are consequently liable to be starved. There is one part of the periphery, however—namely, the medulla, the seat of life itself—which cannot be starved without placing the whole organism in imminent danger.

We may, then, with some degree of impunity have high blood-pressure, but we cannot, without serious menace to life, have anything in the nature of a consistently low blood-pressure. Instances of acute low blood-pressure are afforded by surgical shock and collapse; and certain moderate degrees of sustained low blood-pressure will be found in wasting diseases, such as phthisis, Addison's disease, malignant disease, and the like. There is apparently no limit to the height to which the blood-pressure may rise; at any rate, there is no limit which need concern us. Such limits as exist are presented by the very considerable powers of resistance in the arterial system, for the danger to be anticipated from sudden high pressure is obviously rupture. Long-continued high pressure gives rise to certain definite changes in the blood-vessels in various parts of the body; but these have already been considered, and I do not propose to revert to them.

So much then for recapitulation. To understand the variations of blood-pressure we must recall another physiological fact, scarcely less important than that upon which I have so often insisted —namely, that when there is what our forefathers used to call a determination of blood to a part, as, for instance, in the case of the stomach after a meal, there must necessarily be a definite alteration in the ordinary conditions of blood-pressure. The stomach demands an increased supply of blood for its functional activity, and the organism must supply that increment in such a way that the other parts of the periphery are not unduly depleted. Now, how is this effected? In order to supply the stomach with blood the arteries in the gastric area dilate. If it were not for some compensatory mechanism this would inevitably cause a fall in the mean blood-pressure; that is to say, the pressure in the rest of the periphery would sink below normal. Co-incidently, then, with the dilatation of the gastric arteries there takes place a contraction in some of the other arteries of the body. Parenthetically, it may be remarked that such is the probable explanation of the old adage: "If you eat till you're cold, you will live to grow old." The compensatory contraction of the cutaneous arteries gives rise to a feeling of chilliness.

The arrangement by which this compensation is effected is such that the dilatation of the vessels in one particular area is balanced, not by a general constriction of all the other arteries, but by a constriction of the arteries in another particular area. The reason of this is that the control of the arteries in any given district is vested, not in the medulla, but in the accessory centres situated in the spinal cord, and the balancing of pressures as between two given areas is a matter for these centres alone. The power of the medulla is limited to the production of contraction or dilatation *en bloc*, so that it is only when the ordinary balance is in danger of breaking down that it intervenes to protect itself. In good health, of course, this compensatory or balancing process of the spinal centres is unperceived by the individual; but in departures from good health the patient becomes conscious of symptoms which clearly point to a disturbance of blood distribution in the parts remote from the functioning organ, and this disturbance it is which causes the symptoms.

Still taking as our instance the dilated gastric arteries during the digestive process, when the balance is less accurately adjusted than it ought to be, the constrictive response on the part of the corresponding spinal centre may be inadequate, and a general fall in pressure becomes imminent. The medulla, therefore, interferes by contracting the arteries *en bloc*, with the result that a general rise of blood-pressure ensues, and it is to this general rise that some of the symptoms are due. The want of balance may express itself in the opposite direction. That is to say, instead of the compensatory constrictive response being so inadequate as to necessitate the interference of the medulla, it may be excessive and call forth an area of vaso-dilatation elsewhere. This it is which explains the flushed face and coloured nose of the dyspeptic.

Another, and perhaps a better, instance of a normal process giving rise, under adverse circumstances, to troublesome symptoms is presented by the pelvic organs during menstruation. These organs during that process demand an increased supply of blood; the local arteries accordingly dilate, and the compensatory mechanism in some

* See "Clinical Journal," November 28, 1906, December 5, 1906.

other part or parts is immediately set in motion. In normal healthy menstruation this is unattended by any appreciable rise of blood-pressure. In the menstruation of the would-be fashionable woman of to-day, who squeezes not only her waist but the whole of her abdominal viscera so that her outline shall resemble as far as possible that of a crescent moon, normal conditions no longer obtain, and we find in consequence that during the menstrual period she suffers from symptoms which, though they may vary considerably, are all due to a want of proper balance between the blood-pressure in the pelvic area and that elsewhere. This fact, which is not so well recognised as it ought to be, has a very important bearing on the question of the treatment of these conditions—a matter to which I shall return presently.

Where the starting-point is clearly defined, as it is in the foregoing instances of slight departures from the orderly working of a physiological process, these variations and their consequences are comparatively easy to follow. There are, however, a great number of conditions which, though we may know them to be characterised by variations in blood-pressure, are so obscure in their causation that they appear to be dictated by mere caprice on the part of the vaso-motor apparatus, which would seem to amuse itself by violently contracting one set of vessels and adjusting the balance by a vigorous relaxation of another set, for the purpose, as it were, of keeping its hand in. We know, of course, that these exercises on the part of the governing system are produced by some peripheral irritant acting reflexly, but in a large number of cases that irritant is extremely difficult to find. To account for these cases we are obliged to fall back upon the rather unsatisfactory, but seemingly altogether inevitable hypothesis, that those who are subject to these ebullitions have so unstable a nervous system that they react immoderately to normal stimuli.

The best instance of this kind of vaso-motor disturbance is certainly spasmodic asthma. Whatever the academic doubts affected by the text-books as to the cause of the asthmatic paroxysm, the real, the clinical cause is congestion and turgescence of the vessels in the mucosa of the bronchial tubes. For some reason there is a dilatation of the vessels in this area, which is immediately compensated for by vaso-motor constriction elsewhere; or, it may be, and it is on the whole more probable, that the vaso-constriction elsewhere is the primary event, the dilatation of the bronchial vessels being merely compensatory. At any rate, the vessels in the pulmonary mucosa distend and the asthmatic paroxysm is set in motion. Now, although the asthmatic attack is due to a vaso-dilatation in the bronchial vessels, the proper treatment consists in reducing the vaso-constriction which is balancing this dilatation. And the best, if indeed it be not the only reliable method which is at present open to us for reducing that constriction is to bring about as widespread a vaso-dilatation as we can, so that we may catch in our net, so to speak, the offending constricted area. Because if we can ensure a widespread dilatation of the arteries all over the body, there will be no room for a congestion in any one part. With the channels all patent, the blood will naturally find the paths of least resistance, and the turgescence in the bronchial mucosa will at once be relieved. That is why iodide of potassium and nitrite of amyl, both of which are vaso-dilators, have proved themselves to be our two best symptomatic remedies in cases of spasmodic asthma. I ought, perhaps, to remind you here that these bronchial vessels belong to the systemic and not to the pulmonary circulation. Except that the pressure in the systemic vessels must always be greater than that in the pulmonary, the two systems are altogether independent; in the sense that an area of high pressure in one system cannot be balanced by an area of low pressure in the other.

And what is true of asthma is true also of hay fever or hay asthma. Here, instead of the lower, it is the upper air passages which are afflicted with this congestive turgescence. If, then, we can induce a widespread vaso-dilatation, we relieve the one as certainly as we have relieved the other. But in the case of the upper air passages there is another course open to us which has of late found many enthusiastic advocates, that, namely, of directly provoking a vaso-constriction in the affected mucosa. This can, of course, be brought about by the use of adrenalin spray. By this means we act locally on the offending vessels, and cause them to contract instead of, as in the case of asthma, influencing them indirectly by withdrawing the blood into other channels. Such a course, however, involves a considerable strain on the heart, and it is for this reason a method which is not altogether free from objection.

Another very good instance of this apparently lawless vaso-motor disturbance is afforded by the condition which Hale White has described under the name of "gastrostaxis."[1] We are all familiar with the numberless cases with symptoms closely resembling those of gastric ulcer, which are liable to occur in young women.[2] Hale White has shown that a large proportion of those cases are not gastric ulcer at all; that the hæmatemesis is really due to an oozing of blood from the gastric mucosa in people who have otherwise very little the matter with them, except, perhaps, a moderate degree of chlorosis. Now, this oozing of blood is undoubtedly due to abnormal vaso-dilatation in the gastric mucosa, accompanied by so great a vaso-constriction elsewhere that the pressure in the capillaries of the stomach is unduly raised. This kind of disturbance used by the older writers frequently to be referred to as a kind of vicarious menstruation—a fact which suggests that the hæmatemesis was liable to occur at the time of the period, and was therefore coincident with disturbances of ordinary blood-pressure. Now these cases, when they are mistaken for gastric ulcer, as they almost invariably are, are given astringents such as gallic acid, hammamelis, bismuth, and the like, with a view of checking the hæmorrhage. This has the same effect as we have seen to be produced by the application of adrenalin spray in cases of hay asthma. The balance of pressure is readjusted by producing vaso-constriction in the vessels of the gastric mucosa. A better way would be to reduce the local vaso-dilatation by the other method to which I have referred, namely, that of producing so widespread a vaso-dilatation as to include in the net the constriction, wherever it may be, which is compensating the dilatation in the gastric area.

I may remind you that hæmorrhages of all sorts, notably the hæmoptysis dependent upon phthisis, have recently been very successfully treated by nitrite of amyl, and the rationale of this treatment is, of course, the same as that to which I have just referred—namely, the production of a vaso-dilatation over a large area. The channels in this area being opened up, the pressure of blood in the injured lung is immediately reduced, with the result that the hæmorrhage ceases. When this method of treatment fails, as it occasionally does, it means that the blood has been effused from the vessels of the pulmonic circulation, and not from the ramifications of the bronchial arteries. Inasmuch, however, as the blood-pressure in tuberculosis is as a rule too low, I do not think that it is well to use vaso-dilators in the treatment of hæmoptysis unless the attack is one of very exceptional urgency. Moreover, if reduction of blood-pressure is the real method of cure in a particular case, that cure will come about without outside assistance, because the hæmorrhage itself will effect the reduction.

Ordinary fainting attacks, those, namely, which occur apart from recognisable cardiac disease, are also due to vagaries of blood-pressure. These attacks are generally admitted to be caused by anæmia of the brain, which anæmia is believed by Savill[3] to result, in some cases of hysterical women at any rate, from considerable vaso-dilatation in the splanchnic area. That is why, in conditions of faintness, experience has taught us that the best method of treatment is to empty the splanchnic vessels by the recumbent posture, and to increase the action of the pulmonary suction pump by the inhalation of ammonia and other stimulants. The application of cold to the face and friction to the surface further disturbs the vicious distribution of pressure, and thus assists in the restoration to normal conditions.

There are, however, some forms of syncope which are not so easily explained as this. That, for example, which follows fright or other mental shock. These attacks are explained by Francis Hare in a most instructive paper,[4] as due to a cutaneous vaso-constriction which is so pronounced and widespread as to inhibit cardiac action, and he goes on to say that in no other way does it seem possible to explain the instantaneous effect of nitrite of amyl in many syncopes.

It would be easy to multiply instances of these vagaries of blood-pressure, especially if we touch upon those in which pain is the sign which proclaims that there is a disturbance in progress. For we must remember that these conditions which we have been considering have this in common with pain, that they are nothing more than symptoms. The disturbance in blood-pressure is the mechanism by which the symptoms are provoked, and though by altering the mechanism we may be able to quell the symptoms, we have done nothing to prevent their recurrence. To effect this we must go more deeply into the matter and discover what it is that has caused the disturbance of blood-pressure. Such an investigation leads us at once into the difficult and thorny paths of the central nervous system, whither we cannot at present follow it. We must therefore content ourselves with a consideration of the more prosaic, but not less important, question of the means at our disposal for affording that relief which some of these cases so urgently demand.

To revert, then, to our first instance, that of the active stomach after a meal. Functional dyspepsia, as we know, is either sthenic or asthenic. In asthenic dyspepsia the amount of blood supplied to the stomach is insufficient, the result being that the amount of pepsin and hydrochloric acid is below that which is necessary for adequate digestion. The successful treatment of these cases is directed not only to the artificial supply of pepsin and hydrochloric acid, but also to the increase of blood in the functioning organ by such substances as will increase the local blood supply. No association is more common in my experience than that of asthenic dyspepsia and asthma. It is as if the blood properly belonging to the stomach were, by some caprice, diverted into the bronchial vessels, so that if we have happily succeeded in alleviating the spasmodic ebullition, the state of the digestive power should engage our attention. Sthenic dyspepsia, on the other hand, is due to the fact that too much blood is sent to the organ, with the natural consequence of a congestive turgescence of the mucosa and an excessive secretion of acid. In this condition there is, of course, an area of vaso-constriction elsewhere, and, as we have seen, this vaso-constriction makes itself felt even in cases of normal digestion by that feeling of chilliness to which I have already referred. Now, if this vaso-constriction is excessive, it is reasonable to suppose that nature herself tries to bring about a relieving vaso-dilatation in other parts, and such, I presume, is the cause of the flushing of the face, the running of the nose, and the salivation which we so often see in these cases. Excessive vaso-dilatation in the stomach is compensated for by an excessive vaso-constriction elsewhere, which in its turn may demand for its compensation a dilatation of the vessels of the face; and it is not difficult to believe that this circle should extend its circumference into other districts. The success of the treatment which experience has taught us to adopt in these cases, that namely of giving bismuth and alkalies is, therefore, obvious. The alkalies neutralise the excessive acidity, and the bismuth, while soothing the irritated mucous membrane, constricts the local vessels, and thus contributes to the relief of the symptoms.

The treatment of menstrual disturbances is usually confined to the exhibition of ergot on the one hand, and of bromide of potassium or valerian on the other. Ergot is successful because it acts on the uterus in the same way as we have seen adrenalin spray to act on the upper air-passages, namely, by causing a constriction of the vessels whose abnormal dilatation is the cause of the symptoms. Ergot is, however, very far from being an ideal remedy even in menorrhagia, and its routine administration in pelvic troubles generally, especially in those which accompany the climacteric, is much to be deprecated. For although the drug has a special affinity for the uterine vessels, it undoubtedly causes a contraction of the arteries all over the body. Now, we have seen that the general blood-pressure in menstrual disturbances is already unduly high, so that it is evident that the exhibition of a powerful vaso-constrictor will tend very materially to aggravate the patient's condition even if it does not aggravate her symptoms. For what happens in such circumstances is that the heart is subjected to a very considerable strain, and I am convinced that a great number of the cardiac disorders which occur at or about the menopause are due to the augmentation of hyper-tension (which is usual at such times) by the thoughtless or routine administration of ergot. A better, because a safer and more scientific, way of dealing with menstrual disturbances is to produce vaso-dilatation in other parts, even though we may be aware that the means at our disposal are such that their effects will be transient only. It seems to be a fact that a vicious circle, once broken, does not reconstruct itself readily, so that if we can bring about a widespread vaso-dilatation we may feel fairly confident that any particular attack will thereby be terminated.

Of the methods open to us for bringing about a general relaxation of the arteries, certain drugs are well known to be highly efficacious. Pre-eminent among them stands nitrite of amyl, which is so well known that anything more than a reference to it would be superfluous. Its effect, though very transient, is very decided, and if we would save unnecessary alarm we ought not to neglect the direction that the patient should assume the recumbent posture before inhaling it. Nitro-glycerin and trinitrin both produce the same effect as nitrite of amyl. Inasmuch as they are taken by the mouth, this effect is rather longer in appearing, but it passes off almost as rapidly. Erythrol tetranitrate is a very useful remedy. By its means when given in tabloids of ½ grain every four hours, the arteries may be kept in a state of comparative relaxation for a considerable period. It has one drawback, however, which is that it is liable to cause headache, and patients will sometimes refuse to continue the drug on that account. Iodide of potassium, though most valuable in reducing blood-pressure, is not rapid enough in its action for our present purposes.

In addition to drugs, there are several physical methods of causing a widespread vaso-dilatation which we do well to remember. Foremost among them is venesection, a measure which prejudice on the part of the public prevents us from employing in a large number of cases in which it would confer immense benefit. I have seen it afford immediate relief in spasmodic asthma, and Dr. McDougall, of Cannes[5] enumerates several other spasmodic affections, including croup, in which he has employed it with conspicuous and almost unvarying success.

The action of the ordinary warm bath in effecting dilatation of the arteries should not be forgotten. In cases of emergency it is invaluable, and if it be followed by rest in a thoroughly warmed bed, the vaso-dilatation may be prolonged for a considerable time. Superior even to the hot bath is the hot wet pack, the best method of giving which I have elsewhere[6] described in detail. These means act chiefly upon the cutaneous vessels, it is true, but in a large majority of cases the thorough relaxation of these vessels is sufficient to cause that modification of the blood-pressure distribution which it is our object to effect. When the matter is not one of urgency the radiant-heat baths to be obtained at the Dowsing Institutes are, in addition to being very efficacious, generally much appreciated by patients. The application of high frequency is said to produce definite arterial relaxation. I have, however, no experience of its use. Massage, when really well administered, is of the utmost value in subacute and chronic cases. By this means, not only the cutaneous vessels, but also those in the underlying muscles, are thoroughly dilated and remain so for a considerable period. The combination of hot air and massage, such as we find in a well equipped Turkish bath, is most useful. The objection to the majority of these institutions, namely, that they are imperfectly ventilated, is, however, from our present standpoint, almost insuperable. In conclusion let me say a word of warning against the indiscriminate use of Nauheim-baths in cases where the blood-pressure distribution is faulty. They may do good; in well selected cases they often do; but they have an undoubted and insufficiently recognised capacity for very serious harm.

[1] The Lancet, November 3, 1906. [2] "Are Not some Patients said to be Afflicted with Gastric Ulcer really Suffering from a Different Disease?" Lancet, June 29, 1901. [3] Clinical Journal, May 25, 1904. [4] "Mechanism of the Therapeutic Action of Nitrite of Amyl," Clinical Journal, August 29, 1906. [5] "On the Remedial Value of Blood-letting," American Journal Medical Sciences, July 1887. [6] "Minor Maladies and their Treatment," Baillière, Tindall, and Cox, page 176.

